# Genotypes of cancer stem cells characterized by epithelial-to-mesenchymal transition and proliferation related functions

**DOI:** 10.1038/srep32523

**Published:** 2016-09-06

**Authors:** Chueh-Lin Hsu, Feng-Hsiang Chung, Chih-Hao Chen, Tzu-Ting Hsu, Szu-Mam Liu, Dao-Sheng Chung, Ya-Fen Hsu, Chien-Lung Chen, Nianhan Ma, Hoong-Chien Lee

**Affiliations:** 1 Institute of Systems Biology and Bioinformatics, Department of Biomedical Science and Engineering, National Central University, Zhongli, 32001, Taiwan; 2 Department of Radiation Oncology, Landseed Hospital, Taoyuan, 324, Taiwan; 3 Department of Surgery, Landseed Hospital, Taoyuan, 324, Taiwan; 4 Department of Nephrology, Landseed Hospital, Taoyuan, 324, Taiwan; 5 Department of Physics, Chung Yuan Christian University, Zhongli, 32023, Taiwan; 6 Center for Dynamical Biomarkers and Translational Medicine, National Central University, Zhongli, 32001, Taiwan

## Abstract

Cancer stem cells (CSCs), or cancer cells with stem cell-like properties, generally exhibit drug resistance and have highly potent cancer inducing capabilities. Genome-wide expression data collected at public repositories over the last few years provide excellent material for studies that can lead to insights concerning the molecular and functional characteristics of CSCs. Here, we conducted functional genomic studies of CSC based on fourteen PCA-screened high quality public CSC whole genome gene expression datasets and, as control, four high quality non-stem-like cancer cell and non-cancerous stem cell datasets from the Gene Expression Omnibus database. A total of 6,002 molecular signatures were taken from the Molecular Signatures Database and used to characterize the datasets, which, under two-way hierarchical clustering, formed three genotypes. Type 1, consisting of mainly glia CSCs, had significantly enhanced proliferation, and significantly suppressed epithelial-mesenchymal transition (EMT), related functions. Type 2, mainly breast CSCs, had significantly enhanced EMT, but not proliferation, related functions. Type 3, composed of ovarian, prostate, and colon CSCs, had significantly suppressed proliferation related functions and mixed expressions on EMT related functions.

Genome alteration is a major cause of growth in cancer cells and an important source of genome alteration is mutation. Potential causes of mutation include externally or internally induced DNA damage leading to mutations in protein coding genes, abnormal oncogene activation, and loss of activity in suppressor-genes. An aggregation of such mutations can lead to the formation of cancer cells, and possibly to tumor formation^1^.

Cancer induced mortality has been declining steadily over the past decade primarily due to earlier detection, adjuvant therapies, and the advent of targeted therapies. The sometime disappointing results from standard treatments that include chemotherapy and radiotherapy for the prevention of cancer relapse have recently been attributed to the stem cell-like properties of some cancer cells.

The hypothesis that some cancer cells may have stem cell-like properties was first put forward by Furth and Kahn in 1937, who showed that a single transplanted leukemic cell was able to transmit the systemic disease to a mouse^2^. This hypothesis did not receive substantial support until 1994, when it was shown that only select purified tumor cells with specific markers from acute myeloid leukemia (AML) patients could induce cancer when transplanted into a mouse^3^. Importantly, the cancer-inducing cells with specific markers had stem cell-like self-renewing ability.

Later it was noticed that the cancer-inducing cells tended to be drug-resistant. Only a minute fraction of tumor cells have the conjoined properties of self-renewal, drug-resistance, and cancer-inducement. Recently, such cells have been commonly referred to as cancer stem cells (CSCs). Following a growing number of reports on CSCs in multiple types of cancers, the CSC hypothesis is receiving increasingly wider acceptance^4^,^5^.

The conjoined properties mentioned above is sufficient to set CSCs apart from non-stem-like cancer cells (NSCs), or normal cancer cells^6^. Because NSCs constitute an overwhelming majority of cells in a tumor, they, but not CSCs, are the natural targets of conventional therapy. This may explain why typical cancer therapy is usually ineffective on CSCs. Some attribute cancer recurrence to the drug resistant of CSCs^7^,^8^.

Epithelial-to-mesenchymal transition (EMT) is an essential process in metazoan embryogenesis and is classified into three subtypes: subtypes 1 and 2 are thought to mainly involve the functions of embryogenesis and tissue regeneration, and subtype 3 is associated with gains in malignancy in carcinoma cells^9^. There is increasing evidence showing that cancer cells can be transformed into CSCs through EMT, such as in breast cancer^10^ and prostate cancer^11^. An effective strategy that may lead to tumor remission could be a CSC targeted therapy combined with conventional therapy and a program to block EMT in order to prevent NSCs from becoming CSCs^12^.

CSCs have been identified through biological markers – CD44+ and CD24−13 for breast CSC, CD44+, CD24+, and epithelial-specific antigen (ESA)+^14^ for pancreatic CSC, and C133+^15^ for colorectal CSCs, through selection of a side population from cancer cells during fluorescence-activated cell sorting (FACS)^16^, and by deriving CSCs from spheroids in a cancer cell culture grown with specific culture medium^17^. The advancement of CSC identifying technology has led to an increase in publically available gene expression microarray data on CSCs and cancer cells^18^. To our knowledge, a systematic meta-analysis of this body of data has not been made.

Here we have conducted a comprehensive analysis of publically available 14 gene expression datasets on CSCs versus their NSC counterparts. Four high quality non-stem-like cancer cell and non-cancerous stem cell datasets were used as controls. This CSC data comes from a variety of tissues, including breast, glia, colon, lung, ovary, and prostate. Questions we would like to answer include: what are the differences in the genomic and molecular profiles of CSCs and NSCs? Are these differences tissue-specific? What are the functional characteristics of the differences? We used individual gene-based analysis (IGA) and get set-based analysis (GSA) to study the CSC datasets and found GSA to be the more useful approach. IGA is the standard approach in which a set of genes that are significantly differentially expressed in test versus control samples is used to represent the genomic difference between the two samples. In GSA, that difference is represented by the enrichments of molecular signatures in the difference of the samples. For this study 6,002 molecular signatures from MSigDB were used to query the datasets. Our results indicate that in functional genomic terms the 14 CSC (versus NSC) datasets fell into three genotypes. Type 1, mainly glia CSCs, was found to be significantly enhanced proliferation, and significantly suppressed epithelial-mesenchymal transition (EMT), related functions. Type 2, mainly breast CSCs, had significantly enhanced EMT, but not proliferation, related functions. Type 3, consisting of ovarian, prostate, and colon CSCs, had significantly suppressed proliferation related functions and mixed signatures on EMT related functions.

## Results

### Eighteen datasets with high PCA scores were selected and DEGs extracted

After screening by Principal Component [Fig f1]Analysis (PCA, Methods), 18 CSC microarray datasets were selected (Table 1) for our study, and their differentially expressed genes (DEGs) extracted (Methods). Publically available CSC datasets with low PCA scores were left out. Generally, with the same gene-selection criteria, high-scoring datasets yielded larger DEG sets than low-scoring datasets (Supplementary Fig. 1). However, depending on the inherent difference in the genomic profiles of control and test datasets, the size of DEG varies noticeably even among all datasets having the maximum PCA scores of 1, that is, when control and test datasets are cleanly separated. Of the 18 CSC datasets used 14 had PCA scores of 1 (Table 1), of the remaining four, the dataset Glioma_CD133_GSE37120 had the lowest PCA score 0.75, which means that 3 out of the total 12 datasets (6 control and 6 test) were out of place. This dataset yielded 1400 DEGs, about medium among accepted datasets. Subsequent classification correctly clustered it among other Glioma datasets (Fig. 2). The same applies to the other three datasets with PCA score less than 1 (0.93, 0.92, 0.83; Table 1). Several datasets in the next level of PCA quality (PCA score of 0.50) had been included in early runs but seriously failed classification tests and were excluded in our subsequent analysis.

### DEGs of 18 datasets were converted into molecular signature scores

The DEGs of each dataset were converted to a set of 6,002 normalized enrichment scores (NESs, with corresponding *p*-values), one for each of the 6,002 molecular signatures selected from MSigDB (Methods). The idea of using NESs of molecular signatures instead of expression strengths of DEGs to represent a genomic profile is to better reflect the fact that, in a cell, genes, instead of operating singly and individually – the basic assumption of IGA – operate in groups (here represented by the genes-sets of molecular signatures) to carry out specific functions – the implicit assumption of GSA. This work may be partly viewed as tests of the relative merits of GSA and IGA assumptions. The complete sets of NESs and *p*-values are stored at the online digital downloadable repository Figshare: https://figshare.com/s/34f162ebf4fc02e74a23.

### Different datasets share relatively few common high variance DEGs

To construct uniform representations of the datasets, for each dataset 152 genes with the highest variance across datasets were selected in IGA, and fixed numbers of the most significantly enriched/depleted molecular signatures (152 signature for GSEA^19^, 148 for PAGE^20^, and 167 for GAGE^21^) were respectively selected in three GSA algorithms (Methods). An IGA overlap matrix element between two datasets is the ratio of the size of the intersection of two DEG sets to the size of one of the sets. The overlap matrix is not symmetric; in the lower (upper) triangle the denominator is the row-heading (column-heading) DEG set. The vast majority of the off-diagonal matrix elements of the IGA overlap matrix (Supplementary Fig. 2) are small, less than 0.15, and many are in single digits. The only strong exception is the overlap between hES and iPS. This suggests that IGA will not yield a good classifying of the datasets. The GSA overlap matrices have much larger off-diagonal elements (not shown).

### One-way clustering in GSA, but not IGA, cleanly bipartitions datasets

**Figure 1 f1:**
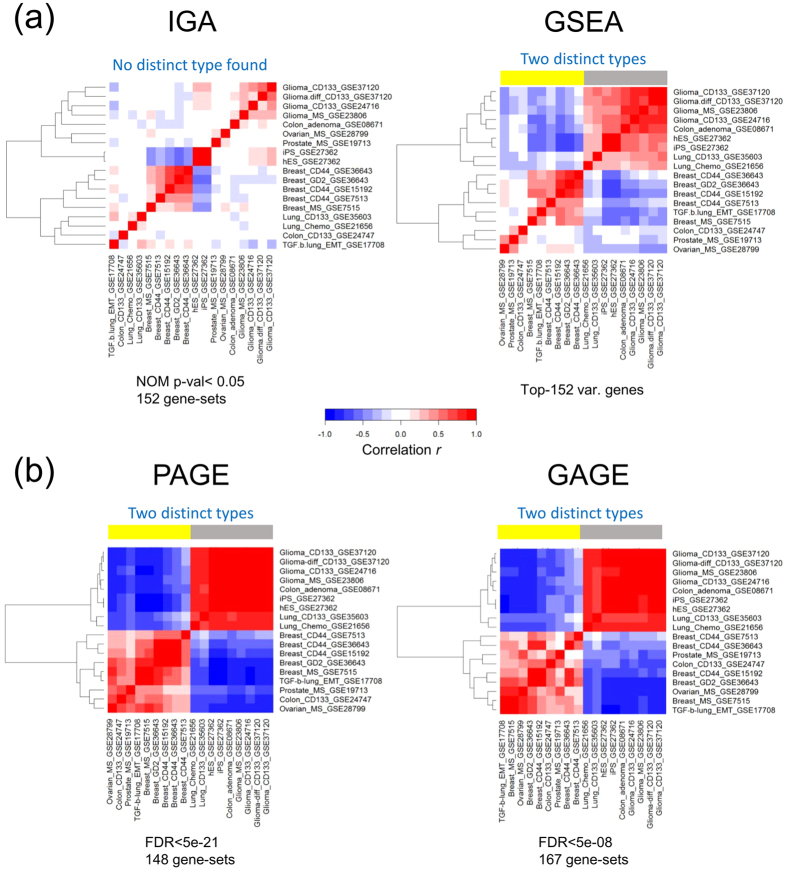
One-way clustering of 18 datasets. (**a**) Left, in IGA, dataset represented by log2-ratios of 152 genes with the highest variance across datasets. Right, in GSEA, dataset represented by NESs of 152 most significantly enriched/depleted signatures (nominal p < 0.05 by GSEA algorithm). (**b**) Left, in PAGE, dataset represented by NNSs of 209 most significantly enriched/depleted signatures (FDR < 5e-18). Right, and GAGE (right), dataset represented by NESs of 197 most significantly enriched/depleted signatures (FDR < 5e-7). Clustering was based on Pearson correlation matrix (Methods).

One-way clustering of datasets based on the Pearson correlation matrix (Methods) yielded different results in IGA and GSA. IGA shows poor clustering; the two large groups are formed at small Pearson correlations (IGA, Fig. 1a). Except for three small groups respectively composed of the doublet iPS and hES, two of four glia, and four of five breasts, all other datasets appear as essentially singles. In comparison the GSA algorithm GSEA^19^ separated the datasets into two distinct large groups formed at relatively large Pearson correlations (Fig. 1a). One group includes the 4 glia CSC and 2 lung CSC, and three control datasets, hES, iPS and colon-adenoma. A second group is composed of the 5-set breast CSC dataset, the three “others” single-set CSC datasets, ovarian, prostate and colon, and the control dataset TGF-b-lung_EMT. The two other GSA algorithms, PAGE^20^ and GAGE^21^, yielded similar results (Fig. 1b).

### GSEA based two-way hierarchical clustering yield three signature clusters and three genotypes

**Figure 2:**
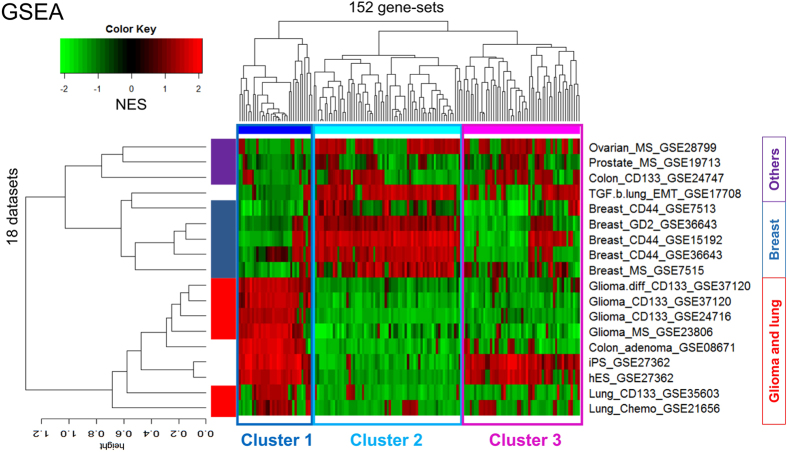
Two-way hierarchical clusters of 18 datasets in GSEA. The heatmap is obtained from the two-way hierarchical clustering of the 18 datasets against the 152 most significantly enriched/depleted signatures from MSigDB (nominal *p* < 0.05 by GSEA algorithm). The 152 signatures form three clusters, Cluster 1, 2, and 3. The 18 datasets also form three clusters, called Type 1 (glia and lung), Type 2 (breast), and Type 3 (colon, prostate, and ovary, or “others”) in the text.

Two-way hierarchical clustering, or heatmap, based on Pearson correlations of log2-ratio of the 152 DEGs in the 18 datasets (Methods) did not yield a useful classification of the datasets (IGA, Supplementary Fig. 3). This could be, as the heatmap shows, because many of the 152 genes exhibit relatively low significance with respect to most datasets. In sharp contrast, most signatures exhibit high significance in the GSEA heatmap based on NESs of the 152 selected signatures (GSEA, Supplementary Fig. 3), which yielded three signature clusters – Cluster 1, 2, and 3 (C1, C2, and C3), containing 33, 67, and 52 signatures, respectively – and three genotype groups, Type 1 (T1), identical to the first group of the last section, Type 2 (T2), composed of sets in the second group except the “others”, and Type 3 (T3), consisting of the three “others” – ovarian, prostate and colon (Fig. 2). Essentially all 33 C1 signatures are over-represented in T1 and under-represented in T2 and T3. The 67 C2 signatures are under-represented in T1, over-represented in T2, and have mixed representation in T3. The 52 C3 signatures are under-represented in T1 (but are conspicuously over-represented in the two stem cell datasets hES and iPS); one subset of C3 is over-represented in T2 and the other subset under-represented; representation in T3 is again mixed (Fig. 2). T2 and T3 weakly form a larger group mainly on the strength of their unified response to C1. The core of T1 is the four 4 glia CSC sets, which form a tight cluster within T1. The core of T2 is the five breast CSC sets; they are not as tightly clusters as the glia are in T1, and the set GSE7513 is a relative outlier.

### Heatmap constructed using cluster gene sets grouped datasets into three genotypes identical to GSEA grouping

To test whether gene-based clustering is inherently inferior to gene-set-based clustering, cluster gene sets (CGSs) of 94, 164, and 157 genes were selected (Methods) from Clusters 1, 2, and 3, respectively (Supplementary Table 1). The union of the three CGSs has 419 genes, 398 of which appear in the gene list of all 18 datasets. This 398-gene set was used to construct a heatmap for the 18 datasets (Supplementary Fig. 4). The result is essentially the same, and yields the same three genotypes, as that given by GSEA (Fig. 2), but different from that obtained with the 152-DEG set (IGA, Supplementary Fig. 3). Proportionately fewer genes exhibit low significance in the 398-gene set heatmap than in the 152-DEG set heatmap. This suggests that it is the procedure of gene selection by differential expression, not IGA per se, that is responsible for producing inferior classification of the datasets.

### Known cancer and stem cell-related signatures cluster datasets into grouping similar to GSEA

**Figure 3:**
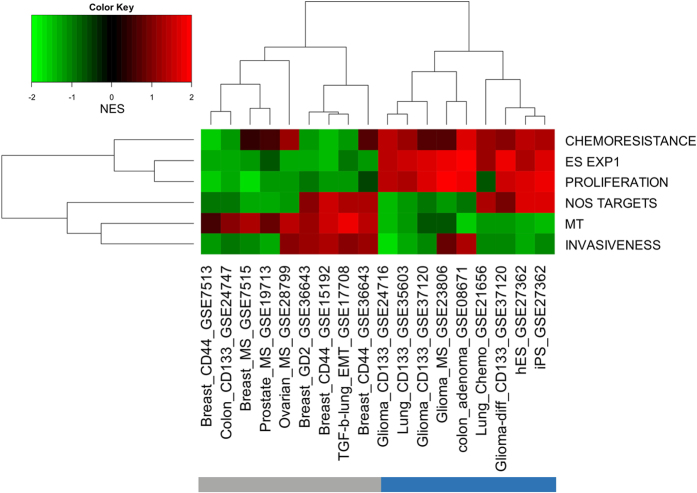
Two-way hierarchical clustering of 18 datasets against six known cancer stem cell related signatures. The six signatures are: chemoresistance [19], ES EXP1 (stem cell) [20], proliferation [21], NOS targets (Nanog, Oct4, SOX2 co-targets) [22], MT sig-nature [23], and invasiveness [24].

Using of six known cancer and stem cell-related signatures – chemoresistance (72 genes^22^), ES EXP1 (stem cell, 40 genes^23^), proliferation (148 genes^24^), NOS targets (179 genes^25^), MT signature (64 genes^26^), invasiveness (186 genes^27^) – to cluster the datasets in the GSA protocol yielded the same two groups seen in Fig. 2. Two among the six signatures, proliferation and MT, could delineate the two groups; proliferation is over-expressed and EMT is under-expressed in group identical T1. The opposite is the case in the group containing T2 and T3 (Fig. 3).

### The three CGSs have distinct Gene Ontology (GO) term enrichments

**Figure 4:**
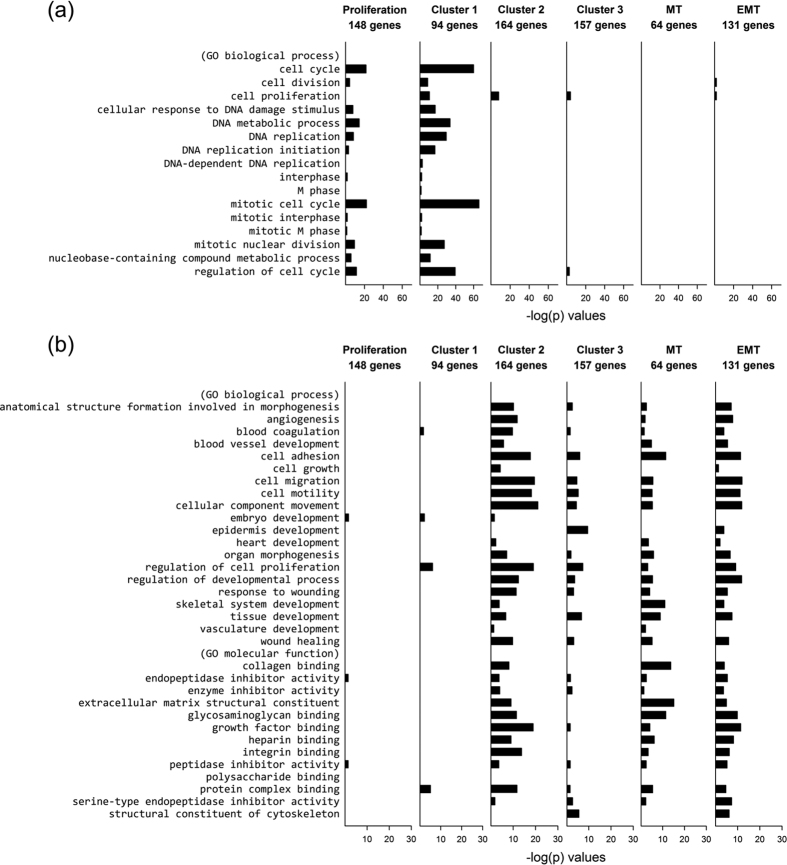
Enrichment of proliferation and EMT related GO terms. (**a**) Enrichment of proliferation related GO terms in the cluster gene sets selected from the signature cluster, Cluster 1, 2, and 3 and in three cancer and stem-cell related signatures: proliferation [34], MT [23], and EMT [25]. (**b**) Enrichment of EMT related GO terms in the same six signatures in (**a**).

The three CGSs, together with the proliferation signature (148 genes [34]) and the MT signature (64 genes^26^) that characterized the datasets (Fig. 3), and an EMT signature (131 genes^28^), were analyzed for gene enrichment in terms of the 16 cancer proliferation-related GO terms^29^ and 33 cancer EMT-related GO terms^28^. The enrichment patterns of the C1-CGS and the proliferation gene signature are similar: enriched in proliferation GO terms (Fig. 4(a)) but not in EMT GO terms (Fig. 4(b)). The enrichment pattern of C2-CGS closely track that of EMT signature and have significant similarity with MT signature; the pattern of C3-CGS broadly follows those of the two EMT-relayed signatures but have smaller −log *p*-values. Neither the C2 nor C3-CGS is enriched in proliferation GO terms (Fig. 4).

### Proliferation and EMT related functions characterize genotypic groups

**Figure 5:**
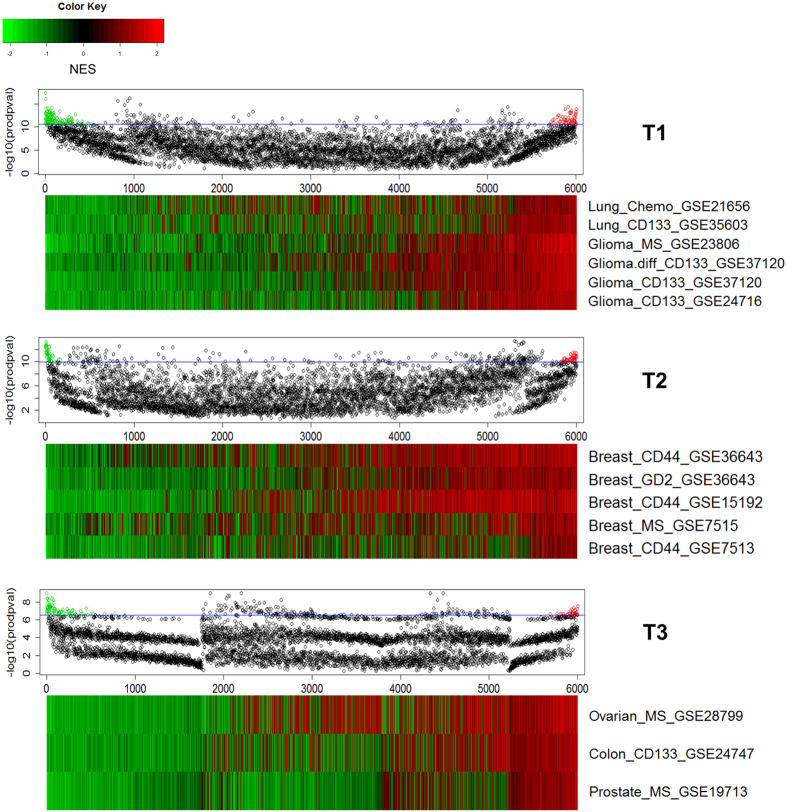
Selection of positive-NES set (PNS) and negative-NES set (NNS) for the three genotypes T1, T2, and T3. The same procedure is applied to each type. Bottom panel (heatmap) in each pair of panels: The 6,002 MSigDB signatures are ranked by NES (for each signature) summed over datasets in the type. Top panel in each pair of panels: Each dot indicates the product of −log *p* values over datasets. Dots above the horizontal line, numbering 300, are those having the top 5% products. Of these 300 signatures, those having positive NES in all the data sets form the PNS (red dots), those having negative NES in all the data sets form the NNS (red dots). The size of PNS/NNS is 58/129 for T1, 48/49 for T2, 37/86 for T3.

**Figure 6:**
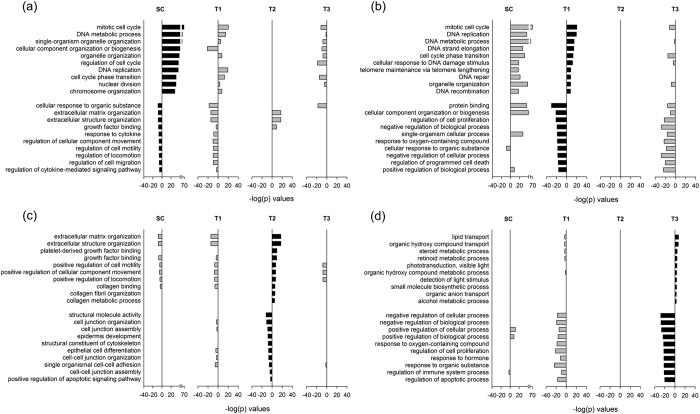
Leading biological process related GO terms in the SC, T1, T2, and T3 genotypes. (**a**) Leading terms in SC (black bars) and their significance in the three other genotypes (gray bars). (**b**) Leading terms in T1. (**c**) Leading terms in T2. (**d**) Leading terms in T3.

Up-regulating genes (URGs) and down-regulation genes (DRGs) were separately selected for the SC, T1, T2, and T3 genotypes (Fig. 5), based on frequency of occurrence in the most significant signatures (Supplementary Fig. 5) (Methods). The URGs and DRGs (Supplementary Table 2) were functionally analyzed in terms of GO terms (Fig. 6; full results at: https://figshare.com/s/6da5e55dfe7ad71e10b8).

The top GO terms of SC are characterized by exceedingly strongly up-regulated terms and weakly down-regulated terms in SC, all are significant in T1, mostly with the same up/down classification; three of the down-regulated terms are up-regulated in T2; all up-regulated terms and two down-regulated terms are down-regulated in T3 (Fig. 6(a)).

The top GO terms of T1 are characterized by strong down-regulated terms and slightly weaker up-regulated terms. Of the 20 top-10 terms (up and down) in T1, all up-regulated terms are also up-regulated terms in SC, one of the down-regulated terms are up-regulated in SC; only one of the up-regulated terms is down-regulated in T2; about half are significant in T3, all down-regulated (Fig. 6(b)).

The GO term analysis of T2 is characterized by moderately moderate up-regulated terms and down-regulated terms. Of the 20 top-10 terms (up and down) in T2, seven of the up-regulated terms are weakly down-regulated in SC; about half are significant in T1, all down-regulated; all except two are not significant in T3 (Fig. 6(c)).

**Figure 7:**
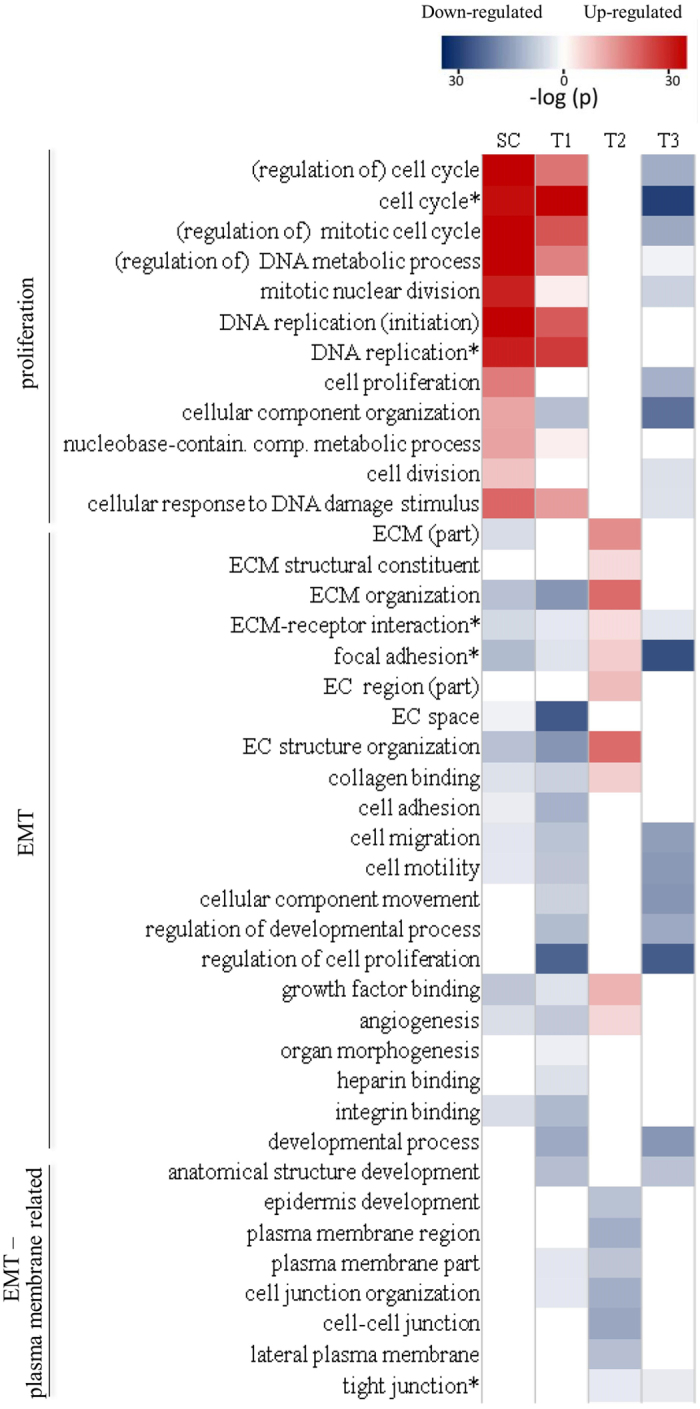
Significant proliferation and MT related GO terms and KEGG pathways in stem cell and the three CSC genotypes. The functional characteristics of the three CSC genotypes are clearly distinct. T1 has high similarity to stem cells (SC): elevated proliferation and suppressed EMT functions. T2 has elevated EMT functions. T3 has suppressed proliferation functions and mixed EMT signatures. Asterisked terms are KEGG pathways.

The top GO terms of T3 are characterized by few weak up-regulated terms and abundant strong down-regulated terms. The 20 top-10 terms (up and down) in T3 are essentially not significant in SC and T2; nine of the down-regulated terms are moderately down-regulated in T1 (Fig. 6(d)). These results are summarized in Fig. 7.

The URGs and DRGs for the SC, T1, T2, and T3 genotypes were also analyzed in terms of KEGG pathways^30^ (https://figshare.com/s/dd2758bc6fdc8d110180). KEGG pathways and GO terms use difference systems of nomenclature. In terms of pathway category, abundance and relation among the four genotypes, the results of KEGG and GO analyses are similar. For instance, KEGG analysis of T3 is also characterized by the few barely significant up-regulated pathways and the many highly significant down-regulated pathways, mostly cancer related. Where directly comparable GO terms and KEGG pathways analyses are in agreement (Fig. 7).

## Discussion

IGA based on DEGs and GSEA based on enriched molecular signatures are fundamentally different approaches to genome representation. Our studies of the classification of the 18 CSC dataset showed GSEA to be the better and more robust approach (Supplementary Fig. 3). It is the selection of genes through differential expression, rather than IGA itself, that made the difference. The combined CGS gene set, the set of highest frequency of occurrence genes culled from the GSEA signatures, was as good a classifier as the GSEA signatures themselves (Supplementary Fig. 4).

As classified by GSEA, the 14 CSC datasets are composed of three genotypes – T1 (glia-lung), T2 (breast), and T3 (others) – each with distinct functional signatures. This functional classification came from three results: two-way hierarchical clustering of the datasets using six known CSC related signatures; GO analysis of the CGSs extracted from the three gene sets, C1, C2, and C3, used in the GSEA classification; GO analysis of URGs and DRGs extracted from the genotypic groups.

The functional associations of many of the selected URGs/DRGs of the three CSC phenotypes have been reported in the literature. These include among T1-UGRs: CDC6, MCM2-7, MSH2; T1-DRGs: CD44, CD24, TGFBI, FN1; T2-URGs: IGFBP3, IGFBP4, FBN1, MMP2, PDGFRA, TWIST1, VIM, TGFBI, FN1; T2-DRGs: CDH1, ERBB3, KRT5, KRT8, KRT14, KRT18, MUC1, OCLN; T3-URGs: APOE, ABCA1, APOE, ABCA1, NR1H3, PLTP, AGR2; T3-DRGs: BRAF, EGFR, KRA*, MAP2K1, PIK3CA, KRAS, PEA15 (annotations with citations given after Supplementary Table 2). Generally, URGs/DRGs in T1 suggest enhanced proliferation and suppressed metastasis, those in T2 indicate EMT activity, and those in T3 indicate suppression of proliferation, drug resistance, tumorigenesis, metastasis but suppression of EMT.

GO analysis (Fig. 4) of the significant gene sets, C1, C2, and C3, reconciles the results of Figs 2 and 3. There, C1 is seen to be highly consonant with the proliferation signature and not with the MT and EMT signatures; C2, the exact opposite; C3, moderately consonant with the MT and EMT signatures and not with the proliferation signature. Since C1 is highly expressed and C2 highly suppressed in T1, and T2 is the opposite (Fig. 2), these partial results in the heatmap are consistent with Fig. 3: T1 is proliferation elevated and T2 is MT/EMT elevated. The heatmap does not sharply define T3; it says T3 is proliferation suppressed but not necessarily MT/EMT elevated. C3 does not play a first-order classification role in the heatmap.

GO analysis of the SC, T1, T2, and T2 datasets, based on their URGs and DRGs, provides further functional details of these genotypes (Fig. 6). The analysis of SC and T1 reveals that the leading up-regulated biological processes in T1 are almost all proliferation related (all the terms are more significant in SC) (Fig. 6(a,b)). In contrast, leading down-regulated biological processes in T1 are either up-regulated (e.g., protein binding, cellular component organization) or not significant in SC (e.g., regulation of cell proliferation, negative and positive regulation of biological process, regulation of programmed cell death). Thus, tumorous T1-CSC proliferates just like healthy SC, but in a less regulated and controlled fashion. In addition, normal biological processes are not followed in T1-CSC. None of the leading GO terms in T1 are significant in T2; some of the leading up-regulated terms (mitotic cell cycle, cell cycle phase transition, organelle organization) and all the leading down-regulated terms in T1 are down-regulated in T3 (Fig. 6(b)).

The most prominent feature of GO analysis of T2 datasets is the strong up-regulation of extracellular matrix (ECM) related terms (Fig. 6(c)). These are strongly down-regulated in T1 and not significant in T3. High ECM density promotes EMT by weakening cell-cell adhesions^31^. Abnormal ECM dynamics are a hallmark of cancer^32^. Most of the leading down-regulated biological processes suggest compromise of the cell-cell junction or epithelial structure (cell/cell-cell junction organization, cell/cell-cell junction assembly, epidermis development, epithelial cell differentiation), which is part of EMT. Most of the up-regulated terms in T2 are down-regulated in T1 (and SC). Three terms related to cell migration – positive regulation of cell motility, positive regulation of cellular component movement, and positive regulation of locomotion – are also down-regulated in T3.

Leading up-regulated biological process-related GO terms in T3 are mostly metabolic-related, all are only weakly significant; the most significant one, lipid transport, has a −log *p*-value of 7.30 (Fig. 6(d)). In contrast, the leading down-regulated terms all have −log *p*-values greater than 20; they mostly indicate disruption of regulation processes, including cellular, biological, proliferation, and immune system, and of responses, including to hormone and organic substance. All leading T3 down-regulated terms are also down-regulated in T1, none in SC or T2. The significant genes and GO term analysis of T3 are consistent in suggesting T3 to be distinct from T2 and not of EMT type, this despite its grouping with T2 in Fig. 2 as a type that is MT-up and proliferation-down. Proliferation related functions are indeed suppressed in T3, but MT/EMT functions are not elevated. T3 is noticeable in having a preponderance of regulation related functions among its leading down-related term. Nineteen of the top-30 down-regulated turns in T3 are of this type, but none of the top-30 up-regulated terms are. Relatively few of the top-30 terms in T1 and T2 are regulation related (Supplementary Table 3). The inference is that instead of specializing on one class of functions as does T1 (proliferation) and T2 (EMT), T3 CSCs cause general and widespread dysfunction of the cell.

The classification of the CSC datasets in terms of the six CSC-related signature grouping T2 and T3 together as elevated EMT and suppressed proliferation, in opposition to T1 (Fig. 3) happens to be too crude. A detailed analysis of the three genotypes in term of proliferation, MT, and plasma membrane-related GO-term shows T2 and T3 to be clearly distinct (Fig. 7). Plasma membrane-related functions are concerned with maintaining the integrity of the epithelium. Suppression of such functions is a part of EMT. In the more detailed description SC and T1 are highly similar: strong proliferation activation and strong MT suppression (stronger in SC in both cases). In T1, but not in SC, plasma membrane related functions are also mildly suppressed, implying mild tendency in damaging the epithelium. T2 exhibits strong MT and anti-epithelial activities, but is neutral in proliferation. T3 exhibits moderate suppression of proliferation, MT, and epithelial activities.

Tumors of breast, prostate, colon, and ovarian cancers are carcinomas, tumors that originate from epithelial cells, which turned cancerous largely through EMT. There have been reports of a “standard” carcinoma tumorigenesis: elevated EMT activity (that induced metastasis) accompanied by suppression of (hyper-) proliferation^33^,^34^,^35^,^36^. T2 (breast), with strongly elevated EMT and proliferation not significant, seems to approximately fit the “standard” description. This is consistent with breast cancer being a strong source of metastasis. Fitting T3 (prostate, colon, and ovary), with both proliferation and the migration part of MT moderately suppressed, into this description appears more problematic. Here it is important to remember that our analysis is based on the difference between CSC and NSC genomic profiles. Hence one possible interpretation of the T3 result is EMT activity in CSC is not low, but is lower than NSC.

Glioma is a type of sarcoma, which does not originate from epithelial cells and whose tumorigenesis and metastasis are not supposed to be driven by EMT. T1 (glia and lung), with its elevated proliferation and suppression of EMT, is a mirror image of the “standard” carcinoma tumorigenesis, and suggests more a general phenomenon of antagonism between EMT and proliferation in tumorigenesis. This is consistent with glia not being known as a source of metastasis. Because lung cancer is a type of carcinoma and is a known source of metastasis, the inclusion of lung CSC in T1 may seem puzzling. A possible explanation is that NSC is a stronger driver of EMT-induced tumorigenesis than CSC in lung. More interesting are the recent reports that EMT is not required in metastasis in lung^37^ and in pancreatic^38^ cancers, both carcinomas.

To summarize, we used gene-set-base analysis to classify 14 CSC datasets into three genomic types, T1 (glia, lung), T2 (breast), T3 (ovarian, colon, prostate), and showed that the three types were characterized by activity level (relative to NSC counterpart) of the two most important cancer related biological functions, EMT and proliferation (Fig. 7): strong proliferation activity of proliferation and moderate suppression of EMT in T1, strong EMT activity in T2, moderate proliferation and EMT suppression in T3.

## Materials and Methods

### Public CSC datasets

We collected available gene expression CSC datasets for multiple cancer types cultured with markers and spheroids methods from the Gene Expression Omnibus (GEO http://www.ncbi.nlm.nih.gov/geo/) database^39^. After quality screening using the Principal Component Analysis (PCA), fourteen CSCs and four control datasets were selected for the study (Table 1)^40^,^41^,^42^,^43^,^44^,^45^,^46^,^47^,^48^,^49^,^50^,^51^. Each CSC dataset consisted of tissue specific gene expression microarray data from CSC and NSC cells. Tissue types included breast, glia, colon, lung, ovary, and prostate. Control data were colon adenoma versus normal colon tissue; human embryonic stem cell (hES) versus fibroblast; induced pluripotent stem cell (iPS) versus fibroblast; and TGF-b EMT versus non-EMT from the lung (Table 1).

### Public databases Molecular Signatures Database (MSigDB)

Version 3.1 of this database has a collection of 8,513 molecular, annotated gene sets divided into 6 major collections: C1, positional gene sets; C2, curated gene sets; C3, motif gene sets; C4, computational gene sets; C5, GO (Gene Ontology) gene sets; C6, oncogenic signatures^52^.

### Workflow

The downloaded CSC and control gene expression datasets (Table 1) were quality screened via Principal Component Analysis (PCA). Datasets (by dataset we always mean a matched pair of sample and control data) were characterized in terms of their genomic profiles in an individual gene approach (IGA, see below) or molecular profiles in a gene set approach (GSA, see below). In GSA, unless otherwise stated, the molecular signatures (henceforth simply signatures) were the 7,998 annotated signatures (C2~C5) in MSigDB^52^. Three types of sample classification were conducted. In the first type, done only in IGA, each dataset was represented by a vector of the percentage overlaps of differentially expressed genes DEGs with itself (100%) and with those of other datasets, and classification was done by clustering the vectors using Pearson distance metric. In the second type, datasets were represented by their genomic (IGA) or molecular (GSA) profiles and were two-way hierarchically clustered using Pearson distance metric. In the third way, done only in GSA, datasets were represented by only signatures that had known CSC signatures and were then two-way hierarchically clustered using Pearson distance metric.

### Quality control by PCA

Principal Component Analysis (PCA) is a statistical procedure that uses an orthogonal transformation to convert a set of vectors based on specific variables into a set of transformed vectors of linearly uncorrelated variables, called principle components (PCs), ranked by their variances. We used PCA to quantitatively express whether the test group and the control group of samples are cleanly separated. Only datasets where the two groups are well separated (PCA score not less than 0.75) were used in the analysis. All data analysis was conducted in R environment (version 2.11.0).

### Individual Gene Approach (IGA) and differentially expressed genes

The IGA approach uses DEGs to represent the genomic difference between the test and control samples. Here, DEGs were selected using the Linear Models for Microarray Data (LIMMA)^53^, based on Student’s t-tests, and fold change analysis. Here, unless otherwise stated, the threshold values for false discovery rate (FDR) <0.05 and fold change (FC) >2 were used.

### Gene Set Approach (GSA) and enrichment scores for molecular signatures

Recognizing the fact that genes do not operate singly in the cell, but rather in groups to carry out specific function, GSA uses differentially enriched molecular signatures to represent the genomic difference between the test and control samples. For this study, we limited signatures that have not less than 10 and not greater than 200 genes overlapping with genes in the microarray gene list, across all microarray platforms involved in the dataset. This reduced the 7,998 signatures (C2~C5) in MSigBD to 6,002. For every dataset, each signature is represented by its enrichment score (ES), normalized according to Gene Set Enrichment Analysis (GSEA)^19^. Normalization accounts for differences in signature size and allows the normalized enrichment scores (NESs) to be compared across all signatures. For comparison purposes we also use two other gene-set methods, Parametric Analysis of Gene Set Enrichment (PAGE)^20^ and Generally Applicable Gene Set Enrichment (GAGE)^21^ on some of the results. Computation in GSEA is carried out using tools provided by the GSEA software website^54^. Those in PAGE and GAGE are carried out using the R package “gage” downloaded from the Bioconductor website^55^.

### Selection of DEGs (in IGA) and signatures (in GSA) for clustering

In IGA, datasets are represented by log2-ratios of 152 genes with the highest variance across datasets. In GSA, datasets are represented by NESs of the most significantly enriched/depleted signatures (in GSEA, 152 signatures determined by those having a nominal *p*-value < 0.05 in not less than 10 of the 18 datasets; in PAGE, 148 signatures determined by FDR <5e-21; in GAGE, 167 signatures determined by FDR <5e-08).

### Classification of datasets by one-way and two-way clustering

For the 18 datasets, an 18 × 18 (symmetric) matrix of pair-wise (i.e., dataset versus dataset) Pearson correlations was computed. Each dataset was represented by the log2-ratios of the selected genes in the case of IGA, and by the NESs of selected signatures in the case of GSA. Then the 18 columns of the matrix were used to construct one-way clusters of the 18 datasets. The 18 × 152 matrices of 18 datasets versus the 152 selected genes (IGA) and 152 selected signatures (GSEA) were use to compute IGA and GSA two-way hierarchical clusters, or heatmaps, respectively. Clustering was carried out using the software package “R”^56^.

### Selection of cluster gene sets (CGS) from datasets

The GSEA two-way clustering of datasets based on 152 molecular signatures yielded three signature clusters, Cluster 1, 2, and 3, each containing 33, 67, and 52 signatures, respectively (Results). For each cluster, a CGS was constructed by taking the union of GSEA leading-edge genes of every signature against every dataset, and by selecting the genes with the highest 5% frequency of occurrence in the signatures.

### Classification by known CSC Signatures

Using the GSEA method we performed an additional GSA classification of the CSC dataset using six known cancer or stem cell-related signatures: Chemoresistance^22^, ES EXP1 (stem cell)^23^, Proliferation^24^, NOS targets (Nanog, Oct4, SOX2 co-targets)^25^, MT signature^26^, and Invasiveness^27^.

### GO and KEGG analyses

The Gene Ontology (GO) is a database that standardizes the representation of gene and gene product attributes across species and databases^57^. In particular, GO gives lists of gene sets known to be associated with specific biological functions, known as GO terms. We used the Heterogeneity-corrected Transcriptome Analysis (HTA) GO extension^58^ and fisher exact test^59^ for conducting GO enrichment analysis.

The Kyoto Encyclopedia of Genes and Genomes (KEGG) is a “computer representation of the biological system”^30^. KEGG Pathway is a collection of maps for cellular and organismal functions (http://www.genome.jp/kegg/pathway.html). We used the database WebGestalt^60^ (http://bioinfo.vanderbilt.edu/webgestalt/) for conducting KEGG pathways enrichment analysis.

### Selection of up-regulated genes (URG) and down-regulated genes (DRG)

The 18 CSC datasets were classified by two-way clustering in the GSA/GSEA protocol into three types – Type 1, Type 2, and Type 3 (Results), and for each type we constructed separate up-regulated genes (URGs) and down-regulated genes (DRGs) for functional analysis, as follows. (a) For each type, only CSC, but not control, datasets were considered. (b) For each dataset in a type, rank the 6,002 MSigDB signatures by NES for the dataset, from positive to negative. (c) For each signature, sum the (GSEA) −log *p* values over the datasets in the type, and select the signatures with the top 5% summed −log *p* values (this will yield 300 signatures). (d) Of the top 5% signatures, keep only those signatures with NES that are either all positive or all negative over the datasets in the type. This will yield a positive-NES set of signatures (PNS), and a negative-NES set (NNS). (e) Do GSEA for each signature in PNS against each dataset in the type, and collect the leading-edge genes^19^. (f) Rank the collected genes by their frequencies of occurrence in PNS, genes in the top 5% as selected to form the URG set. (g) Repeat (e) and (f) on NNS to generate the DRG set.

## Additional Information

**How to cite this article**: Hsu, C.-L. *et al*. Genotypes of cancer stem cells characterized by epithelial-to-mesenchymal transition and proliferation related functions. *Sci. Rep.* **6**, 32523; doi: 10.1038/srep32523 (2016).

## Supplementary Material

Supplementary Information

## Figures and Tables

**Table 1:** The 18 gene expression datasets, PCA scores, and number of DEGs.

Name	Contrast (no. of chips)	Reference	PCA score	DEGs
Breast_CD44_GSE15192	CD44+/CD24−(4) vs CD44−/CD24+(4)	40	1	3093
Breast_CD44_GSE36643	CD44+/CD24−(3) vs CD44−/CD24+(3)	41	1	1137
Breast_CD44_GSE7513	CD44+/CD24−(14) vs non-CD44+/CD24−(15)	42	0.93	1278
Breast_GD2_GSE36643	GD2+ (3) vs GD2−(3)	41	1	465
Breast_MS_GSE7515	MS (15) vs non-MS (11)	42	1	2877
Colon_CD133_GSE24747	CD133+ (3) vs CD133−(3)	N/A	1	247
Glioma_CD133_GSE24716	CD133+ (4) vs CD133−(4)	43	1	606
Glioma_CD133_GSE37120	CD133+ (6) vs CD133−(6)	N/A	0.75	1400
Glioma_MS_GSE23806	MS (17) vs non-MS (12)	44	1	2049
Glioma-diff_CD1d33_GSE37120	CD133+ (6) vs CD133−(6)	N/A	0.83	1291
Lung_CD133_GSE35603	CD133+ (3) vs CD133−(3)	45	1	5591
Lung_Chemo_GSE21656	cisplatin-resistant (3) vs cisplatin-senstive (3)	46	1	123
Ovarian_MS_GSE28799	MS (3) vs non-MS (3)	47	1	2100
Prostate_MS_GSE19713	MS (6) vs non-MS (6)	48	0.92	618
Colon_adeno ma_GSE08671	ade (32) vs nor (32)	49	1	1927
hES_GSE27362	hES (3) vs fbs (3)	50	1	3106
iPS_GSE27362	iPS (8) vs fbs (3)	50	1	3060
TGF-b-lung_EMT_GSE17708	EMT (3) vs non-EMT (3)	51	1	2279
