# Supplementary material for: Genotypes of cancer stem cells characterized by epithelial-to-mesenchymal transition and proliferation related functions
Source: Sci Rep. 2016 Sep 6;6:32523. doi: 10.1038/srep32523 (PMC5011650; doi:10.1038/srep32523)
Supplement: Supplementary Information [file srep32523-s1.pdf]

Genotypes of cancer stem cells characterized by epithelial-to-mesenchymal transition and proliferation related functions

Chueh-Lin Hsu[+1], Feng-Hsiang Chung[+1], Chih-Hao Chen[1], Tzu-Ting Hsu[1], Szu-Mam Liu[1], Dao-Sheng Chung[2], Ya-Fen Hsu[3], Chien-Long Chen[4], Nianhan Ma*[1], and Hoong-Chien Lee*[1,5,6]

[1]Institute of Systems Biology and Bioinformatics, Department of Biomedical Science and Engineering, National Central University, Zhongli, Taiwan 32001

[2]Department of Radiation Oncology, Landseed Hospital, Taoyuan, Taiwan 324

[3]Department of Surgery, Landseed Hospital, Taoyuan, Taiwan 324

[4]Department of Nephrology, Landseed Hospital, Taoyuan, Taiwan 324

[5]Department of Physics, Chung Yuan Christian University, Zhongli, Taiwan 32023

[6]Center for Dynamical Biomarkers and Translational Medicine, National Central University, Zhongli, Taiwan 32001

[+]CLH and FHC made equal contributions.

*Corresponding authors. HCL: hclee12345@gmail.com; NM: nianhan.ma@gmail.com.

Supplementary Information

Suppl. Fig. 1

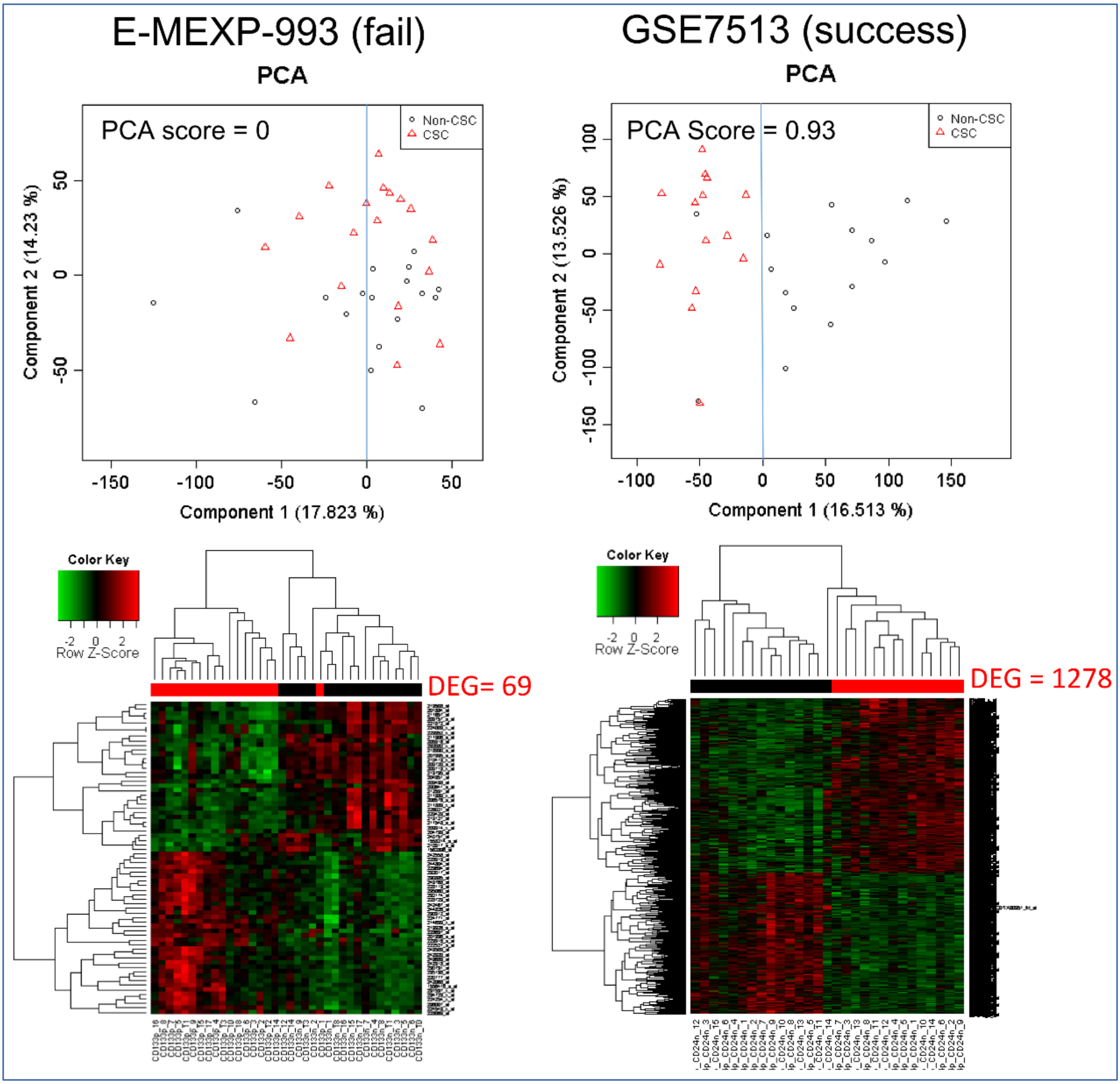
E-MEXP-993 (fail)
GSE7513 (success)
PCA
PCA score = 0
PCA Score = 0.93
Non-CSC
CSC
Component 1 (17.823 %)
Component 2 (14.23 %)
Component 1 (16.513 %)
Component 2 (13.526 %)
Color Key
Row Z-Score
DEG= 69
DEG = 1278

Supplementary Figure 1 High PCA-scoring datasets yielded larger differentially expressed gene (DEG) sets than low-scoring datasets

The dataset E-MEXP-933 had a PCA score of zero and yielded 69 DEGs. The dataset Breast_CD44_GSE7513 had a PCA score of 0.93 and yielded 1278 DEGs. Genes were selected using LIMMA [51], with false discovery rate < 0.05 and fold change > 2.

Suppl. Fig. 2

DEG overlap(%)	**Breast_CD44_GSE15192 (3093)**	**Breast_CD44_GSE36643 (1137)**	**Breast_CD44_GSE7513 (1278)**	**Breast_GD2_GSE36643 (465)**	**Breast_MS_GSE7515 (2877)**	**Colon_CD133_GSE24747 (247)**	**Glioma_CD133_GSE24716 (606)**	**Glioma_CD133_GSE37120 (1400)**	**Glioma_MS_GSE23806 (2049)**	**Glioma-diff_CD133_GSE37120 (1291)**	**Lung_CD133_GSE35603 (5591)**	**Lung_Chemo_GSE21656 (123)**	**Ovarian_MS_GSE28799 (2100)**	**Prostate_MS_GSE19713 (618)**	colon_adenoma_GSE08671 (1927)	hES_GSE27362 (3106)	iPS_GSE27362 (3060)	TGF-b-lung_EMT_GSE17708 (2279)
**Breast_CD44_GSE15192 (3093)**	100	62	28	63	24	9.3	9.2	11	16	11	15	6.5	21	17	13	7.2	6.7	26
**Breast_CD44_GSE36643 (1137)**	23	100	14	64	13	2.8	3.3	3.6	6.3	4	6.4	1.6	9.9	6.6	5.2	2.9	2.6	10
**Breast_CD44_GSE7513 (1278)**	12	16	100	20	9	2.8	5.3	4.9	4.9	4.5	7.6	3.3	8.6	8.6	4.4	3.3	3.5	9
**Breast_GD2_GSE36643 (465)**	9.4	26	7.3	100	4.1	1.2	1	1.3	3.2	1.4	2.6	2.4	3.7	3.4	2.3	1	1	3
**Breast_MS_GSE7515 (2877)**	22	32	20	25	100	8.1	11	8.3	22	8.3	12	5.7	18	20	17	6.2	6.6	20
**Colon_CD133_GSE24747 (247)**	0.7	0.6	0.6	0.7	0.7	100	4	2.9	0.9	1.9	0.7	1.6	0.6	0.5	0.9	0.6	0.6	1
**Glioma_CD133_GSE24716 (606)**	1.8	1.8	2.5	1.3	2.3	9.7	100	18	2.1	14	2.1	2.4	1.5	1.6	2.4	1.5	1.2	1.7
**Glioma_CD133_GSE37120 (1400)**	4.9	4.4	5.4	3.9	4	16	41	100	5.3	47	4.6	5.7	4.2	4.5	5.1	3	2.9	4.3
**Glioma_MS_GSE23806 (2049)**	11	11	7.9	14	16	7.3	7.1	7.8	100	7.4	9.6	6.5	11	14	22	4.3	5.1	11
**Glioma-diff_CD133_GSE37120 (1291)**	4.5	4.6	4.5	3.9	3.7	9.7	29	44	4.7	100	4.1	2.4	3.9	4.4	5	2.9	2.7	3.9
**Lung_CD133_GSE35603 (5591)**	27	32	33	32	24	16	19	18	26	18	100	13	30	29	27	12	12	34
**Lung_Chemo_GSE21656 (123)**	0.3	0.2	0.3	0.7	0.2	0.8	0.5	0.5	0.4	0.2	0.3	100	0.3	0.3	0.4	1	1.3	0.4
**Ovarian_MS_GSE28799 (2100)**	14	18	14	17	13	5.3	5.3	6.4	11	6.3	11	5.7	100	21	12	5.2	4.9	11
**Prostate_MS_GSE19713 (618)**	3.3	3.6	4.2	4.5	4.3	1.2	1.7	2	4.3	2.1	3.2	1.6	6.2	100	4.5	1.3	1.3	2.7
colon_adenoma_GSE08671 (1927)	8.1	8.9	6.7	9.7	11	6.9	7.8	7.1	21	7.4	9.3	5.7	11	14	100	3.7	4.4	12
hES_GSE27362 (3106)	7.2	7.8	8.1	6.9	6.7	7.3	7.4	6.7	6.5	7.1	6.8	24	7.7	6.6	5.9	100	81	7.1
iPS_GSE27362 (3060)	6.7	7	8.4	6.7	7	7.7	6.3	6.4	7.6	6.4	6.8	32	7.2	6.6	6.9	80	100	7.3
TGF-b-lung_EMT_GSE17708 (2279)	19	20	16	15	16	8.9	6.4	7.1	12	6.8	14	6.5	12	10	14	5.2	5.4	100

Supplementary Figure 2 Percentage overlap matrix of DEG sets selected from 18 datasets

DEGs for each dataset was selected using LIMMA, with FDR < 0.05 and FC >2. An entry in the upper (lower) triangle of the overlap matrix is the ratio of the number of common genes to number of genes in DEG sets of corresponding dataset in the top row (column on left).

Suppl. Fig. 3

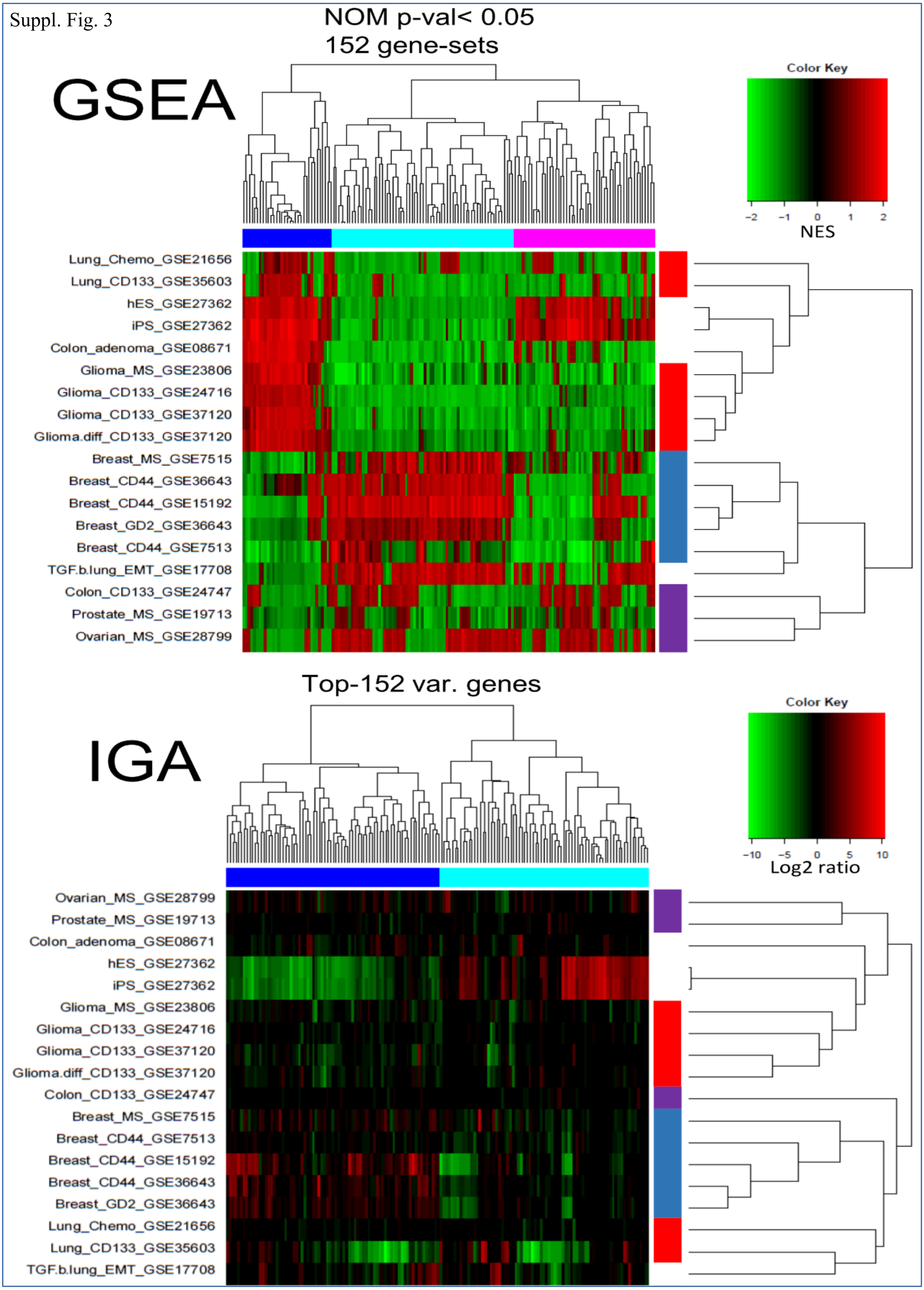
NOM p-val< 0.05
152 gene-sets
GSEA
Color Key
−2 −1 0 1 2
NES
Lung_Chemo_GSE21656
Lung_CD133_GSE35603
hES_GSE27362
iPS_GSE27362
Colon_adenoma_GSE08671
Glioma_MS_GSE23806
Glioma_CD133_GSE24716
Glioma_CD133_GSE37120
Glioma.diff_CD133_GSE37120
Breast_MS_GSE7515
Breast_CD44_GSE36643
Breast_CD44_GSE15192
Breast_GD2_GSE36643
Breast_CD44_GSE7513
TGF.b.lung_EMT_GSE17708
Colon_CD133_GSE24747
Prostate_MS_GSE19713
Ovarian_MS_GSE28799
Top-152 var. genes
IGA
Color Key
−10 −5 0 5 10
Log2 ratio
Ovarian_MS_GSE28799
Prostate_MS_GSE19713
Colon_adenoma_GSE08671
hES_GSE27362
iPS_GSE27362
Glioma_MS_GSE23806
Glioma_CD133_GSE24716
Glioma_CD133_GSE37120
Glioma.diff_CD133_GSE37120
Colon_CD133_GSE24747
Breast_MS_GSE7515
Breast_CD44_GSE7513
Breast_CD44_GSE15192
Breast_CD44_GSE36643
Breast_GD2_GSE36643
Lung_Chemo_GSE21656
Lung_CD133_GSE35603
TGF.b.lung_EMT_GSE17708

Supplementary Figure 3 Two-way hierarchical clusters of 18 datasets in IGA and GSEA

Top, in GSEA, dataset represented by NESs of 152 most significantly enriched/ depleted molecular signatures (nominal $p < 0.05$ by GSEA algorithm). Bottom, in IGA, dataset represented by log2-ratios of 152 genes with the highest variance across datasets. Clustering was based on Pearson correlation of log2-ratios in IGA and NESs in GSEA (Methods).

Suppl. Fig. 4

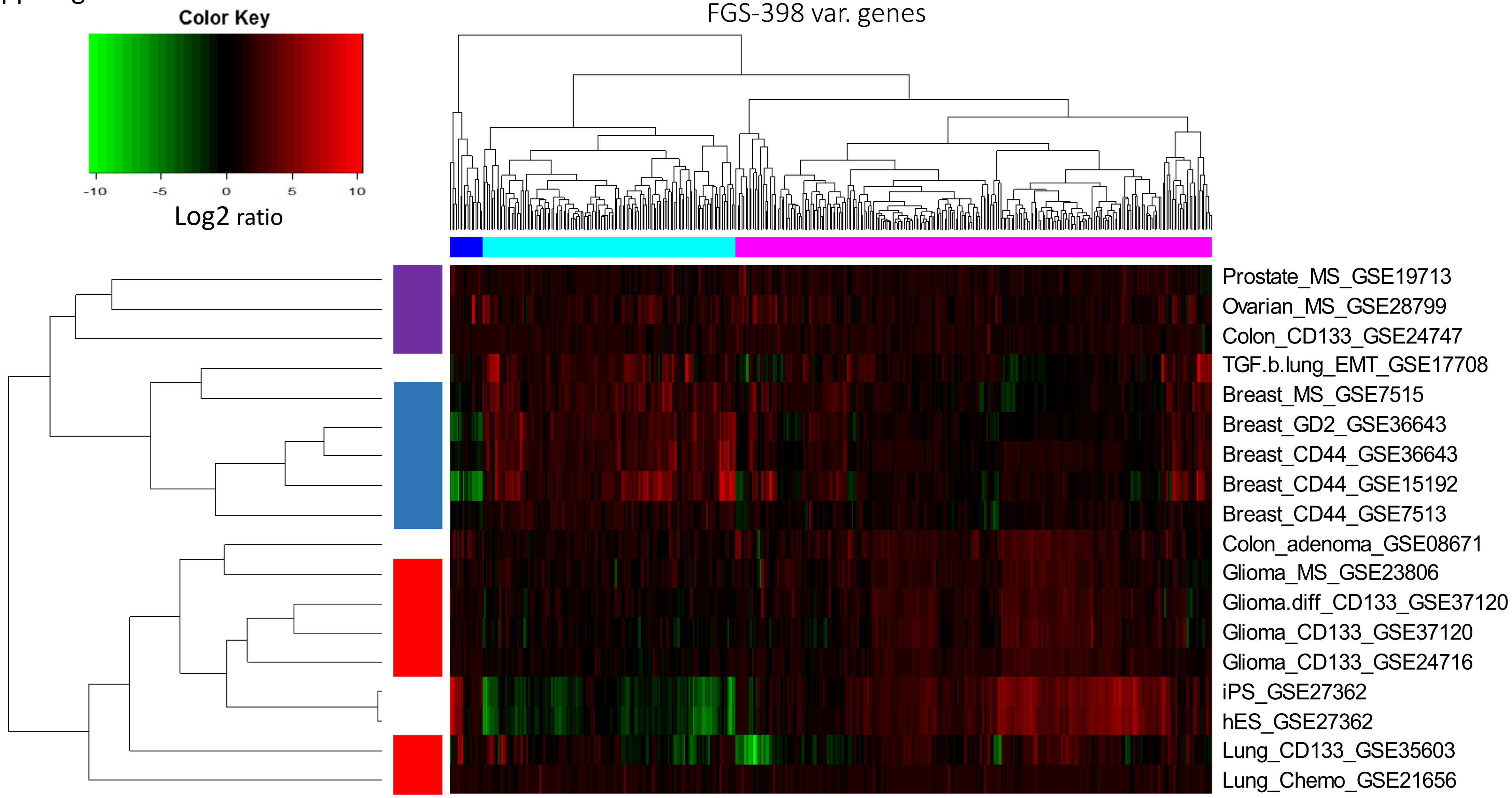
Color Key
-10
-5
0
5
10
Log2 ratio
FGS-398 var. genes
Prostate_MS_GSE19713
Ovarian_MS_GSE28799
Colon_CD133_GSE24747
TGF.b.lung_EMT_GSE17708
Breast_MS_GSE7515
Breast_GD2_GSE36643
Breast_CD44_GSE36643
Breast_CD44_GSE15192
Breast_CD44_GSE7513
Colon_adenoma_GSE08671
Glioma_MS_GSE23806
Glioma.diff_CD133_GSE37120
Glioma_CD133_GSE37120
Glioma_CD133_GSE24716
iPS_GSE27362
hES_GSE27362
Lung_CD133_GSE35603
Lung_Chemo_GSE21656

Supplementary Figure 4 Heatmap for18 datasets against 398 genes selected from 152 signatures

The 398 genes are the highest occurrence frequency (top 5%) genes in the 152 signatures used in the heatmap of Fig. 2. Contents of the three cluster formed by the 18 datasets are identical to those in Fig. 2.

Suppl. Fig. 5

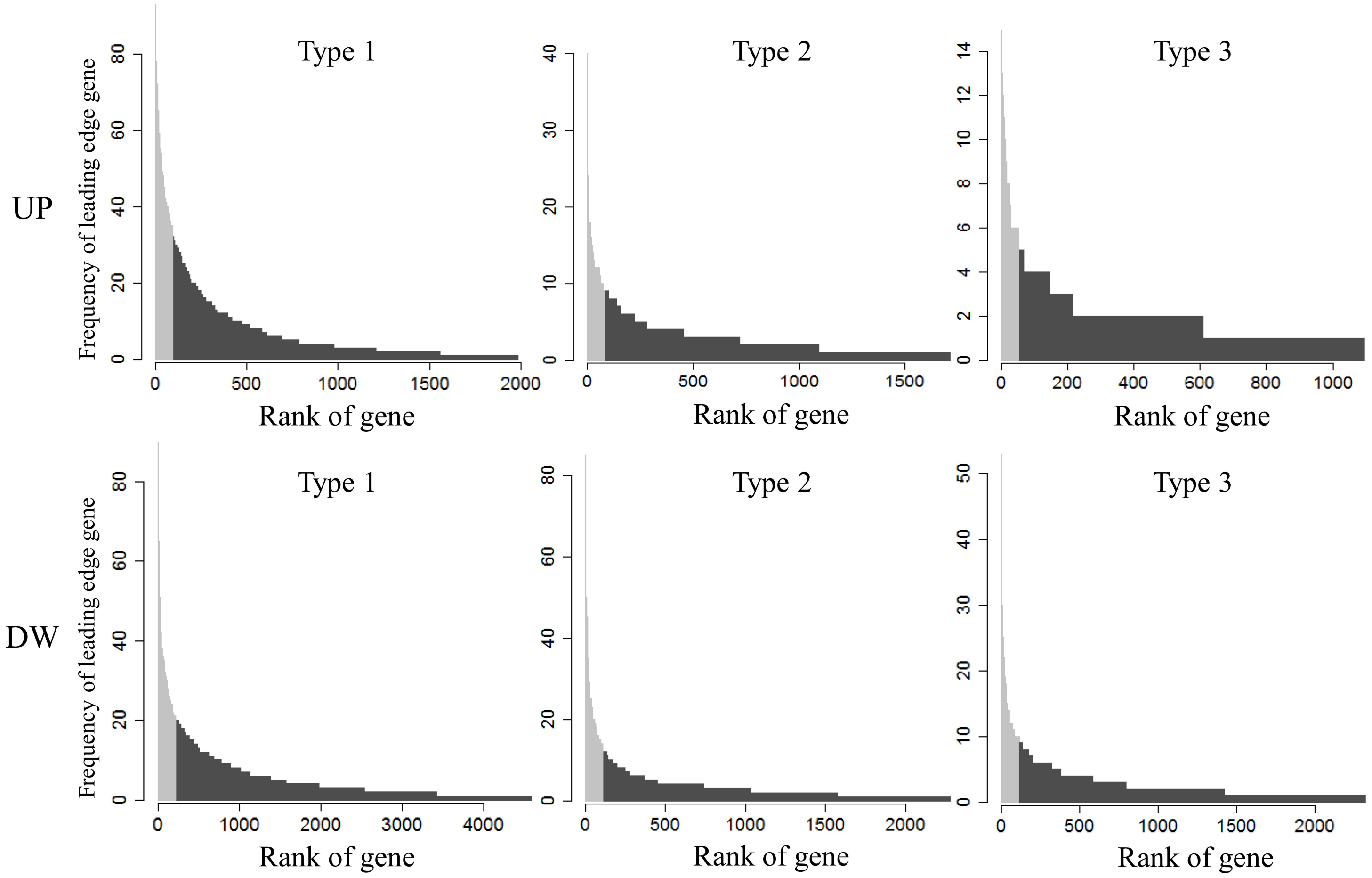
UP
DW
Type 1
Type 2
Type 3
Type 1
Type 2
Type 3
Frequency of leading edge gene
Frequency of leading edge gene
Rank of gene
Rank of gene
Rank of gene
Rank of gene
Rank of gene
Rank of gene
0 500 1000 1500 2000
0 500 1000 1500
0 200 400 600 800 1000
0 1000 2000 3000 4000
0 500 1000 1500 2000
0 500 1000 1500 2000
0 20 40 60 80
0 10 20 30 40
0 2 4 6 8 10 12 14
0 20 40 60 80
0 20 40 60 80
0 10 20 30 40 50

Supplementary Figure 5 Selection of highest occurrence frequency Up-regulated genes (URGs) and down-regulated genes (DRGs) for the three genotypes T1, T2, and T3

Genes in the PNS and NNS signatures (Fig. 5) are separately ranked by frequency of occurrence. Those having the top 5% frequencies in PNS (NNS) are selected as URGs/DRGs. The size of URG/DRG is 83/191 for T1, 70/91 for T2, 46/95 for T3.

Supplementary Table 1 Cluster gene sets (CGS) selected from three signature clusters in heatmap of Fig. 2.

Cluster	Gene list
Cluster 1<br>94 genes	CDC6,CCNA2,CDK2,CDC7,MCM3,MAD2L1,BUB1,AURKA,MCM2,CCNB1,UBE2C,SMC1A,HDAC2,CDK4,KIF11,DBF4,RRM1,MCM4,BIRC5,BUB1B,PKMYT1,CCNB2,SKP2,PCNA,HMGB2,PLK1,CDC20,RAN,MCM6,FEN1,RAD54L,E2F1,MCM5,ESPL1,POLD1,KIAA0101,RAD51,MCM7,TTK,CHEK1,E2F3,CDC25A,ZWINT,SSRP1,TOP2A,MYC,PRKDC,H2AFZ,CDC25C,KHDRBS1,BUB3,SMC3,KIF2C,NOLC1,RFC4,TYMS,CENPF,NASP,POLA1,ANAPC10,TFDP1,TIPIN,STMN1,RACGAP1,TPX2,POLE,LSM4,CDH1,EED,NCAPH,USP1,NUSAP1,CDK6,SCARB1,NME1,SNRPA,CCT3,FBXO5,PRMT5,SMC4,CDC23,KIF22,TCP1,CCT5,CDCA5,CCNE2,CDKN3,NEK2,TIMELESS,ERBB3,RPA3,CCNE1,CSE1L,C1QBP
Cluster 2<br>164 genes	CD44,NRP1,DAB2,PPAP2B,IL1R1,SERPINE1,COL3A1,TGFBI,PDGFRA,TPM1,THBS1,IGFBP3,COL1A2,FN1,S100A4,RRAS,VIM,CYP1B1,LAMB1,TIMP1,SRPX,SPARC,TIMP2,EMP3,CCL2,TNFRSF1A,GAS6,MYLK,TXNIP,ACTA2,BCL6,LUM,COL1A1,CLU,DKK3,C1S,TGFB1,LAMC1,CD59,MMP2,CD63,CDKN1A,GBP2,AKR1C3,COL6A1,MMP1,CYR61,CEBPD,PRSS23,B2M,DLC1,RAB31,SEMA3C,COL6A3,FTL,NNMT,TSC22D3,CTSB,MGLL,CDH11,OSMR,TRAM2,COL5A1,NRCAM,IGFBP5,PDGFRB,AKR1C1,FBN1,PLOD2,HTRA1,PMP22,FTH1,BTG1,ITGAV,POSTN,EPS8,ADM,TSC22D1,MARCKS,COL4A1,CALD1,GABARAPL1,PDE4DIP,LMO2,LOXL2COL6A2,GPNMB,ITGA5,HSPB8,VEGFC,CCDC80,TRIM22,APOE,TPM2,PHLDA1,NUAK1,FAP,LPXN,IL6,ID2,NR3C1,MT1H,S100A6,ITGB5,RGL1,LOX,TAGLN,THBS2,TIMP3,SYNE1,SERPING1,FADS1,CDH2,SEMA5A,LOXL1,SNAI2,CPM,EMP1,ACTN1,CD151,DCN,CCND1,GAS1,LAMB2,EPHX1,GJA1,IL6ST,COL5A2,COL8A1,ACSL1,PCDH9,ZCCHC6,GSN,LRP1,MT1F,ANPEP,CD14,EFEMP1,ENPP2,CDH13,GEM,SMAD3,AHNAK,PAPSS2,SAT1,ANXA1,GLIPR1,ANXA5,SPOCK1,SHC1,ZBTB20,RAB13,RHOBTB3,TGFBR3,MAN1A1,LTBP2,ARL6IP5,IFITM3,IFI16,ENG,STAT6,SEPP1,HLA-F,YPEL5
Cluster 3<br>157 genes	CDH1,CLDN7,KRT18,S100P,SPINT2,CLDN4,CD24,KRT19,SCNN1A,AGR2,MAP7,GATA3,ERBB3,PPL,SLC9A3R1,CA2,TPD52,MUC1,ELF3,VAV3,MAL2,KRT8,SOX4,GRB7,LAMB3,SLPI,MYB,SSH3,LAMC2,PKP3,TJP3,FXYD3,CLU,AQP3,TACSTD2,CLDN1,EGFR,DNAJC12,SORL1,KRT17,TRIM29,MYO5C,AKR1C1,FBP1,CDH3,MOSC1,PDK4,SELENBP1,MAOA,MT2A,KRT15,TPD52L1,FOXA1,S100A2,LAD1,KCNK1,SFN,DSP,JUP,TFAP2C,KRT5,PERP,BTG1,ID2,DDR1,TFPI2,CA12,SDC4,NEBL,ANXA3,RTKN,B3GNT3,AKR1C3,SERPINB5,FGFR3,MT1X,RND1,KRT14,DST,MATN2,KIAA1324,KRT6B,BIK,OCLN,SFRP1,EHF,HOOK1,GPR160,MLPH,ITGB4,MMP7,ST14,MEST,AZGP1,KRT7,GPM6B,GABRP,SOX9,F2RL1,ARHGEF5,CLGN,ARL4C,LLGL2,GALNT3,F11R,ABCG2,TGM2,AKR1C2,IGFBP2,STC1,RAPGEF5,CECAM6,RNF43,PODXL,ETV4,NMU,LPL,LCN2,KLF5,TMEM30B,RAB25,CYB5A,SLC27A2,SYTL2,PRSS23,PHLDA1,SYT17,ANXA9,CAMK2N1,TFF3,SCUBE2,UCP2,BLVRB,WWC1,CNKSR1,CCND1,RHOD,ST6GAL1,ATF3,CHD7,CELSR1,HIPK2,LIMA1,MYO6,GPX3,MYLK,RHOB,STOM,S100A14,FLNB,SPINT1,DFNA5,TMEM45B,MBP,TGFA,KIAA0040,EPN3

Supplementary Table 2 Up-regulated genes (URG) and down-regulated genes (DRG) of SC and the three CSC genotypes T1, T2, and T3. All listed genes are top-5% in frequency of occurrence, those with asterisk attached are top-1%.

Type	Gene list
SC-URG 154 genes	ANLN,ASPM,AURKA,AURKB,BIRC5,BLM,BRCA1,BUB1,CCNA2,CCNB1,CCNB2,CCND2,CCNE1,CCNE2,CCNF,CCT5,CD24,CDC20,CDC25A,CDC6,CDC7,CDCA3,CDCA5,CDCA7,CDCA8,CDH1,CDK2,CDKN3,CENPA,CENPE,CENPF,CEP55,CHAF1A,CHEK1,CKS1B,CKS2,CSE1L,CYCS,DBF4,DKC1,DLGAP5,DNMT1,DTL,E2F8,ECT2,ESPL1,EXO1,EZH2,FBXO5,FEN1,FOXM1,GINS1,GINS2,GMNN,H2AFX,H2AFZ,HAT1,HDAC2,HELLS,HMGA1,HMGB2,HMMR,HNRNPA2B1,HNRNPAB,HNRNPD,HNRNPU,ILF2,KIF11,KIF14,KIF15,KIF18B,KIF22,KIF23,KIF2C,KIF4A,KIFC1,KNTC1,KPNA2,LBR,LIG1,LMNB1,MAD2L1,MCM10,MCM2,MCM3,MCM4,MCM5,MCM6,MCM7,MELK,MKI67,MLF1IP,MSH2,MSH6,MYBL2,NASP,NCAPD2,NCAPH,NCL,NDC80,NEK2,NOLC1,NUSAP1,PAICS,PKMYT1,PLK1,PLK4,POLA1,POLA2,POLD1,POLE,POLE2,PRC1,PRIM1,PTTG1,RACGAP1,RAD51,RAD51AP1,RAD54L,RAN,RANBP1,RFC2,RFC3,RFC4,RFC5,RPA1,RRM1,RRM2,SET,SMC1A,SMC2,SMC3,SMC4,SNRPA,SNRPB,SNRPD1,SNRPE,SNRPF,SOX4,SSRP1,STMN1,TK1,TMPO,TOP2A,TOPBP1,TPX2,TTK,TYMS,UBE2C,UBE2S,UNG,USP1,<br>XPO1,ZWINT
SC-DRG 56 genes	ADM,B2M,BNIP3L,CASP4,CAV1,CCNG2,CD44,CD59,CDKN1A,COL3A1,COL6A1,COL6A3,CYP1B1,CYR61,DAB2,DCN,DST,EGFR,EMP3,GAS6,GLRX,IFIT3,IFITM3,IGFBP3,IGFBP5,IL1R1,IL6ST,JAK1,JAK2,LAMB1,LUM,MMP1,NRP1,P4HA1,P4HA2,PDGFRA,PDGFRB,PIK3CA,PRKCA,S100A4,SERPINE1,SPARC,SRPX,STAT1,STAT2,STAT3,STAT4,STAT5A,STAT6,TGFBI,TGFBR2,THBS1,TIMP1,TIMP3,TYK2,VEGFC
T1-URG 17* + 66 genes	BRCA1,BUB1B,BUB3*,CCNA2,CCT5,CDC23,CDC25A,CDC25C,CDC6*,CDC7,CDK2*,CDK4,CHAF1A,CHEK1,DCP2,DEK,DKC1,DNAJC9,DNMT1,DUT,E2F1,ESPL1,FEN1,H2AFZ*,HAT1,HDAC1,HDAC2,HMGB2,ILF2,ILF3,KHDRBS1*,LMNB1,LMNB2,LSM4,MAD2L1,MCM2*,MCM3*,MCM4,MCM5*,MCM6,MCM7,MSH2*,NASP*,NCL,NONO,PCNA,PLK4,POLA1,POLD1,POLE,PPP1CC,PRKDC,RAD21,RAD23A,RAD51,RAD54L,RBMX,RFC3,RFC4*,RPA1,RRM1*,SERBP1,SET,SKP2,SMC1A*,SMC3*,SNRPA*,SNRPA1,SNRPB,SNRPD1,SNRPD3,SNRPE*,SNRPG,SRPK1,SSBP1,SSRP1,TARS,TCP1,TFDP1,TMPO,TOP2A,USP1*,ZWINT
T1-DRG 39* + 152 genes	A2M*,ABCA1,ABCC3,ABCG1,ABHD4,ACTA2,ACTN1,ADCY7,ADM,AEBP1,AHNAK,AIF1,AKR1C1*,AKR1C3,ANXA2,ANXA4,ANXA5,APOE*,APP,AQP3,ATP1B1,AXL,BCL6*,BMP1,BTG1,BTG2,C1QB,C1S,CAPG*,CASP1,CCL2,CCL5,CD14,CD24*,CD44*,CD59*,CD63,CD9*,CDH11,CDKN1A,CBPD*,CFD,CHI3L1,CLU*,CNN1,CRIP1*,CRYAB*,CTSB*,CTSD,CTSH,CTSS*,CXCL12,CYP1B1,DAB2*,DAPK1,DDR1,DHRS3*,DKK3,DLC1,DUSP1,ECM1,EFEMP1,EGFR,EMP1,EPAS1,EPHX1,FN1*,FOS,GABARAPL1,GALC,GAS6*,GATM,GBP1,GBP2*,GJA1,GLRX*,GM2A,GPNMB,GPX3,GSN,HIST1H2AC,HIST2H2BE,HLA-E,HLA-F,HMOX1,HSPB8,HTRA1*,ID2*,IER3,IFI16,IFITM2,IGFBP5,IL13RA1,IL1R1*,IL32,IL6,IRF7,ITGAM,ITGB2* ,ITGB5*,JUNB,KRT19,KRT7,LAMB1,LAMP2,LGALS1,LGALS3BP*,LILRB2,LITAF,LMO2,LST1,LTBP2,MAOA*,MAP3K8,MGLL,MMP1,MMP7,MT1H,MT2A,MUC1,MVP,MYL9,MYLK*,MYO1F,NAGK,NCF2,NEDD9,NFKBIA,NNMT,NPC2,NRCAM,NRP1*,PBX1*,PDE4DIP,PHLDA1,PLA2G4A,PLAU*,PLAUR,PLOD2,PLTP,PMP22,PPAP2A,PPAP2B*,PPT1,PSAP*,PSMB9,RBP1,RGL1,RHOB,RIN3,RRAS,S100A13,S100A4,S100A6,SAT1*,SCG2,SELL,SEMA3B,SEPP1,SERPINE1,SH3BGRL3,SOCS3,SOD2,SORBS1,SOX4,SPARC,SPINT2,SPP1,SRPX,STOM,TAGLN,TCIRG1,TCN2*,TGFB1,TGFB2,TGFBI,TGFBR2,THBS1,TIMP1*,TIMP2,TMEM127,TPP1,TRIM22,TSC22D3*,TXNIP*,VCAM1,VCL,VEGFC,VIM,VWF,YPEL5
T2-URG 15* + 55 genes	AD-<br>AM12,AKAP12,ANKH,BGN,CCL2,CD99,CDH11*,CFH,COL18A1,COL1A2,COL3A1*,COL4A1,COL6A1,CSF1,CYP1B1*,<br>DAB2,DCN*,DPT,DPYSL2,EFEMP2,ETS1,ETV1,ETV5,EVI2A,F2R,FBN1*,FN1,FYN,GRB10,HLA-G,HSD11B1,HTRA1*,IGFBP3*,IGFBP4*,IGFBP6,INSIG1,KCNAB1,LAPTM5,LOXL2,MMP2*,NDN,NFIX,NID1,NRP1,OLFML3,PCDH9,PCOLCE,PDGFRA*,PDGFRB,PHF17,PIK3R1,PLAT,PLXND1,PMP22*,POSTN*,RASA3,S100A4,SATB1,SERPINH1,SH2B3*,SLC6A6,SPARC,TCN2,TGFBI,THBS2,TIMP2*,TRIB2,TWIST1, VIM*,WNT5A
T2-DRG 18* + 73 genes	AGR2,AIM1,ANK3,AQP3,ARHGEF5,ATP1B1,AZGP1,B3GNT3,BIK,BLNK,CD24*,CD9,CDH1*,CDH3,CEACAM6,CLDN4,CLDN7*,CLU,CRABP2,CXADR,CYB5A,DSC2,DSG2,DSG3,DSP*,EHF,ELF3,EPN3,EPPK1,ERBB3*,F11R*,FOXA1,FXYD3*,GALNT3,GTA3,GMDS,GRB7,HOOK1,IRF6,IRX5,ITGB6,JUP,KIAA0040,KRT14*,KRT15,KRT17,KRT18*,KRT19*,KRT5,KRT6B,KRT7,KRT8*,KRT8P11,LAD1,LAMB3,LAMC2,LLGL2,MAP7*,MUC1*,MYB,MYO5C,MYO6,OCLN*,PERP,PKP3,PPL,PTPRF,S100A14,S100A8,S100P*,SCNN1A*,SFN,SFRP1,SLC9A3R1,SLPI,SOX4,SPINT1,SPINT2*,SSH3,ST14,TACSTD2,TFAP2C,TJP2,TJP3,TMEM30B,<br>TPD52*,TPD52L1,TRPS1,TSPAN1,VAV3,WWC1
T3-URG 10* + 36 genes	AADAC,ABCA1*,ACOX2,ACP5,AGR2*,AKR1C1*,AKR1C2,AKR1C3,APOA1,APOC3,APOD,APOE*,BAAT*,BHMT,CA5A,CD14,CD74*,CEBPA,CES2,CLU,CPT2,CXCL1,CXCL12,CXCL2,DGKA,DPP4,DUSP6*,ETNK2,FADS1,FBP1, HLA-DMA,HLA-DMB,<br>HLA-DQB1,IFI30,IGF1,MASP2,NPR1,NR1H3*,OTC,PCK1,PLTP*,RBP4,RHOB,SELENBP1*,TFF3,TNFAIP2

T3-DRG<br>20* + 75<br>genes	ABLIM1,AKT3,ANAPC10,ANXA1,APC,ATR,AVEN,BARD1,BCAT1,BCL2,BCL2L1,BIRC3,BIRC5,BNIP2,BNIP3,BRAF*,BUB1,CALD1,CASP3,CAV1*,CCNA2*,CCND1,CCNE1,CCNE2,CDC16,CDC23,CDC25B,CDC25C,CDC42EP3,CDC6,CDK6*,CDK8,CDKN1A,CDKN1C,CENPF,CFLAR,CHEK1,CHUK,COL4A2,CRIM1,CUL1,DBF4,E2F3,EGFR*,FLNA,FN1,FYN,GAB2*,GPX1,GRB2*,GSK3B*,GSTP1,HDAC3,HDAC9,HRAS*,HTATIP2,IGF1R,IKBKB,ITGAV,ITGB1,KRAS*,KRT7,MAD2L1,MAP2K1,MAPK1,MT2A,MYC,MYH9,NCK2,NRAS*,PAK2,PEA15*,PFKP,PIK3CA*,PIK3CB*,PIK3CD,PIK3R1*,PIK3R3,PTPN11,PTPN14,RB1,SAMD4A,SHC1*, SKP2,SOS1*,SPTBN1,STAT1,TAX1BP1,TCF4,THBS1,TIPIN,TPM1*,TUBB2A,YWHAH,ZFP161

Annotation of T1, T2 and T3 genes in Supplementary Table 2 Among the T1-URGs, CDC6* is an oncogenic cell-cycle gene [1]; MCM2*, MCM3*, MCM4, MCM5*, MCM6, and MCM7 are marker genes for proliferation [2]; and MSH2* is a DNA repair enzyme gene [3]. Among the T1-DRGs, CD44* and CD24* and markers of breast CSC [4] that indicate enhanced invasive capability [5]; TGFBI promotes metastasis [6]; APOE* (when expressed) indicates enhanced invasive and metastasis capabilities [7]; and FN1* indicates metastatic outgrowth [8]. All of the above suggest enhanced proliferation and suppressed metastasis. Among the T2-URGs, IGFBP3* and IGFBP4* express the presence of cytokines for mesenchymal stem cells [9]; FBN1*, MMP2*, PDGFRA*, and TWIST1 indicate EMT activity [10]; VIM* promotes cell morphogenesis during EMT [11]; TGFBI promotes metastasis [6]; and FN1 indicates metastatic outgrowth [8]. Among the T2-DRGs, CDH1*, ERBB3*, KRT14*, KRT18*, KRT5, KRT8*, MUC1*, and OCLN* are epithelial markers [12-15]. All of these are indications of EMT activity. Among the T3-URGs, APOE* indicates enhanced invasive and metastasis capabilities [7]; ABCA1* indicates resistance to chemo drugs [16]; APOE*, ABCA1*, NR1H3*, and PLTP* indicate cell-migration related [17] cholestorol metabolism [18]; AGR2* indicates tumorigenesis and induces metastasis [19]. Among the T3-DRGs, BRAF*, EGFR*, KRAS*, MAP2K1, and PIK3CA* induce EMT leading to invasion [20,21]; KRAS* also promotes proliferations and suppresses diversification and apoptosis [22]; PEA15* regulates cell's invasive ability [23]. The signals from T3 are mixed; together it indicates suppression of proliferation, drug resistance, tumorigenesis, metastasis but suppression of EMT.

References cited in Supplementary Table 2

1. Malumbres, M. & Barbacid, M. Cell cycle, CDKs and cancer: a changing paradigm. *Nature Reviews Cancer* **9**, 153-166, doi:10.1038/nrc2602 (2009).
2. Blow, J. J. & Hodgson, B. Replication licensing—Origin licensing: defining the proliferative state? *Trends in cell biology* **12**, 72-78, doi:10.1016/S0962-8924(01)02203-6 (2002).
3. Fujii, H. *et al.* Sphere-forming stem-like cell populations with drug resistance in human sarcoma cell lines. *International journal of oncology* **34**, 1381-1386, doi:10.3892/ijo_00000265 (2009).
4. Al-Hajj, M., Wicha, M. S., Benito-Hernandez, A., Morrison, S. J. & Clarke, M. F. Prospective identification of tumorigenic breast cancer cells. *Proceedings of the National Academy of Sciences* **100**, 3983-3988, doi:10.1073/pnas.0530291100 (2003).
5. Sheridan, C. *et al.* CD44+/CD24-breast cancer cells exhibit enhanced invasive properties: an early step necessary for metastasis. *Breast Cancer Res* **8**, R59, doi:10.1186/bcr1610 (2006).
6. Ma, C. *et al.* Extracellular matrix protein βig-h3/TGFBI promotes metastasis of colon cancer by enhancing cell extravasation. *Genes & development* **22**, 308-321, doi:10.1101/gad.1632008 (2008).
7. Sakashita, K. *et al.* Clinical significance of ApoE expression in human gastric cancer. *Oncology reports* **20**, 1313-1319, doi:10.3892/or_00000146 (2008).
8. Soikkeli, J. *et al.* Metastatic outgrowth encompasses COL-I, FN1, and POSTN up-regulation and assembly to fibrillar networks regulating cell adhesion, migration, and growth. *The American journal of pathology* **177**, 387-403, doi:10.2353/ajpath.2010.090748 (2010).
9. Liu, C.-H. & Hwang, S.-M. Cytokine interactions in mesenchymal stem cells from cord blood. *Cytokine* **32**, 270-279, doi:10.1016/j.cyto.2005.11.003 (2005).
10. Lehmann, B. D. *et al.* Identification of human triple-negative breast cancer subtypes and preclinical models for selection of targeted therapies. *The Journal of clinical investigation* **121**, 2750, doi:10.1172/JCI45014 (2011).
11. Mendez, M. G., Kojima, S.-I. & Goldman, R. D. Vimentin induces changes in cell shape, motility, and adhesion during the epithelial to mesenchymal transition. *The FASEB Journal* **24**, 1838-1851, doi:10.1096/fj.09-151639 (2010).
12. Martin, P. *et al.* Prostate epithelial Pten/TP53 loss leads to transformation of multipotential progenitors and epithelial to mesenchymal transition. *The American journal of pathology* **179**, 422-435 (2011).

13. Fuchs, B. C. *et al.* Epithelial-to-mesenchymal transition and integrin-linked kinase mediate sensitivity to epidermal growth factor receptor inhibition in human hepatoma cells. *Cancer research* **68**, 2391-2399, doi:10.1158/0008-5472.CAN-07-2460 (2008).
14. Guaita, S. *et al.* Snail Induction of Epithelial to Mesenchymal Transition in Tumor Cells Is Accompanied by MUC1 Repression andZEB1 Expression. *Journal of Biological Chemistry* **277**, 39209-39216, doi:10.1074/jbc.M206400200 (2002).
15. Samavarchi-Tehrani, P. *et al.* Functional genomics reveals a BMP-driven mesenchymal-to-epithelial transition in the initiation of somatic cell reprogramming. *Cell stem cell* **7**, 64-77, doi:10.1016/j.stem.2010.04.015 (2010).
16. Gillet, J.-P., Efferth, T. & Remacle, J. Chemotherapy-induced resistance by ATP-binding cassette transporter genes. *Biochimica et Biophysica Acta (BBA)-Reviews on Cancer* **1775**, 237-262, doi:10.1016/j.bbcan.2007.05.002 (2007).
17. Zuo, W. & Chen, Y.-G. Specific activation of mitogen-activated protein kinase by transforming growth factor-β receptors in lipid rafts is required for epithelial cell plasticity. *Molecular biology of the cell* **20**, 1020-1029, doi:10.1091/mbc.E08-09-0898 (2009).
18. Legry, V. *et al.* Associations between common genetic polymorphisms in the liver X receptor alpha and its target genes with the serum HDL-cholesterol concentration in adolescents of the HELENA Study. *Atherosclerosis* **216**, 166-169, doi:10.1016/j.atherosclerosis.2011.01.031 (2011).
19. Liu, D., Rudland, P. S., Sibson, D. R., Platt-Higgins, A. & Barraclough, R. Human homologue of cement gland protein, a novel metastasis inducer associated with breast carcinomas. *Cancer research* **65**, 3796-3805, doi:10.1158/0008-5472.CAN-04-3823 (2005).
20. De Roock, W., De Vriendt, V., Normanno, N., Ciardiello, F. & Tejpar, S. KRAS, BRAF, PIK3CA, and PTEN mutations: implications for targeted therapies in metastatic colorectal cancer. *The lancet oncology* **12**, 594-603, doi:10.1016/S1470-2045(10)70209-6 (2011).
21. Berg, M. & Soreide, K. EGFR and downstream genetic alterations in KRAS/BRAF and PI3K/AKT pathways in colorectal cancer—implications for targeted therapy. *Discovery medicine* **14**, 207-214 (2012).
22. Irahara, N. *et al.* NRAS mutations are rare in colorectal cancer. *Diagnostic molecular pathology: the American journal of surgical pathology, part B* **19**, 157, doi:10.1097/PDM.0b013e3181c93fd1 (2010).
23. Glading, A., Koziol, J. A., Krueger, J. & Ginsberg, M. H. PEA-15 inhibits tumor cell invasion by binding to extracellular signal-regulated kinase 1/2. *Cancer research* **67**, 1536-1544, doi:10.1158/0008-5472.CAN-06-1378 (2007).

Supplementary Table 3 Number of regulation related terms in the top-30 up-regulated and down-regulated GO terms in the three CSC genotypes.

Type	T1		T2		T3	
Up or down regulated	Up	Down	Up	Down	Up	Down
Number of terms	0	6	3	2	0	19
Range of –log p	<7.68	16.7-21.0	7.01-7.29	2.99-6.48	<2	18.3-28.0
